# Refeeding Edema in Restrictive Eating Disorders: Beyond Acute Body Weight Gain

**DOI:** 10.1002/erv.70017

**Published:** 2025-07-28

**Authors:** Yosua Yan Kristian

**Affiliations:** ^1^ Institute of Psychiatry Psychology and Neuroscience King's College London London UK

**Keywords:** malnutrition, refeeding edema, refeeding syndrome, restrictive eating disorders

## Abstract

**Background:**

One study recently suggested that phosphate supplementation might contribute to the occurrence of refeeding edema in patients with restrictive eating disorders (EDs) with severe malnutrition complications.

**Objective:**

This commentary aims to provide insight into the study while suggesting a more detailed approach to defining malnutrition, refeeding syndrome, and edema.

**Main Discussion:**

There are several diagnostic criteria for diagnosing malnutrition, some of which might overlap with the criteria of refeeding syndrome risks. A precise nutritional and hydration status assessment is needed before starting nutritional therapy for patients with restrictive EDs. With the potential occurrence of refeeding edema during the refeeding practice in these individuals, this commentary discusses the available assessment methods to differentiate edema and other conditions that are related to acute body weight gain. Furthermore, this commentary also outlines the potential pathomechanism involved and provides future recommendations for studies and clinical practice.

**Conclusions and Implications:**

Understanding the pathomechanism of the development of refeeding edema is important to ensure patient safety during refeeding practices in patients with restrictive EDs. Further studies are needed to understand this complex mechanism, which includes analyzing the involvement of hyperinsulinemia and capillary leakage as a potential etiology of refeeding edema.

## Highlights

- Malnutrition and refeeding syndrome can co‐occur in patients with restrictive eating disorders. A distinction of these differences should be made clear before treatment.
- An acute body weight gain during patient refeeding could not be solely used as a diagnosis for refeeding edema.
- There is a need to accurately determine nutrition and fluid status pre‐, intra‐, and post‐admission in patients with restrictive eating disorders to address refeeding edema.

Dear Editor,

I am writing to comment on the article titled “Association of Phosphate Supplements with Refeeding Edema in Restrictive Eating Disorders” (Khatri et al. 2025). The authors conducted a retrospective cohort study involving 633 patients diagnosed with anorexia nervosa (AN) and avoidant/restrictive food intake disorder (ARFID), both of whom were experiencing severe malnutrition. Among these patients, 247 (39%) received phosphate supplementation either orally or intravenously, depending on their phosphate levels. The authors compared the weight gain of the supplemented group with that of the non‐supplemented group. According to the study, patients who received phosphate supplementation experienced greater acute weight gain. It was suggested that phosphate supplementation may contribute to refeeding edema in patients with restrictive eating disorders (EDs) who are severely malnourished.

Firstly, I would like to highlight the nutritional diagnoses of the patients included. The authors did not describe severe malnutrition, which can be diagnosed in multiple ways, including the most recent diagnosis provided by the Global Leadership Initiative on Malnutrition (GLIM) (Cederholm et al. 2019). Whilst generally severe malnutrition is diagnosed with a body mass index (BMI) less than 16 kg/m^2^, the mean BMI of non‐supplemented patients was 14.1, with a standard deviation of 2.1. This could be interpreted that there were patients with a BMI of 16 or above, which did not represent severe malnutrition. I also appreciate that the authors performed a subgroup analysis for people with a BMI ≤ 13 kg/m^2^. However, the significant risk of refeeding is determined by a BMI < 16 kg/m^2^ (da Silva et al. 2020). Thus, excluding patients with a BMI of 14–15.9 kg/m^2^ might not capture all patients at a significant risk of refeeding.

My second comment arose from the determination of edema. While I agree that acute weight gain is related to water retention, it is important to note that plasma volume depletion is a common finding in severely malnourished patients with AN. In these cases, patients often exhibit hemoconcentration and hypovolaemic hyponatremia. This condition typically improves with fluid supplementation, leading to an increase in body weight, with an average gain of up to 1 kg in 4 days (Caregaro et al. 2005). In such situations, early weight gain accompanied by improved laboratory results might indicate adequate rehydration rather than edema. Furthermore, weight gain alone is not a sufficient indicator of fluid overload. A multiparametric approach, considering clinical symptoms such as heart failure, increased respiratory rate, ascites, and edematous extremities, alongside body composition assessments, imaging, and biochemical results, is necessary to accurately determine whether a patient is experiencing fluid overload (Koratala et al. 2022).

The study showed that most early weight gain occurred in the first 10 days. However, no additional measurements were taken to determine the fluid status in patients. Additionally, I found an interesting pattern in the BMI subgroup analysis, where only patients in the BMI ≤ 12 kg/m^2^ group showed a significant weight gain difference at 21 days, which might need further investigation of why, only at a specific BMI range, the weight gain remained significant in the long term. Exploring their clinical outcomes and biochemistry might shed light on this phenomenon.

Other factors related to the development of edema that might be overlooked were nutrition and fluid intake. Fluid intake would significantly differ between patients prescribed oral, enteral, or parenteral nutrition. Simple fluid monitoring, such as a daily fluid balance chart, could be used to estimate patients' fluid status, although it does not depict edema directly. Therefore, it is crucial to assess a patient's fluid status at admission, during treatment, and prior to discharge before concluding that any weight gain is due to edema.

While some missing links and factors may have been overlooked in the definition and assessment of malnutrition and edema in patients in this study, I am generally interested in the direction of the research by the authors on the pathomechanism of ‘refeeding edema.’ This future research could include a comparison of the occurrence of edema in patients with AN who received nutritional treatment based on current refeeding syndrome guidelines. Theoretically, refeeding edema in severely malnourished patients arises from a sudden increase in nutritional intake, which is why refeeding syndrome guidelines were developed to prevent such complications (da Silva et al. 2020). Investigating the occurrence of refeeding edema in patients with restrictive EDs who have been treated according to these guidelines could provide insights into personalizing care and distinguishing between different clinical conditions.

Another important consideration for understanding the pathomechanism of refeeding syndrome is the exploration of different macronutrient intakes during refeeding. Kohn et al. (2011) highlighted disturbances of insulin and phosphate metabolism as central to this process (Kohn et al. 2011). When glucose is reintroduced during refeeding, it triggers an insulin surge. This surge drives glucose, fluid and electrolytes, including phosphate, into the intracellular space, resulting in a low serum phosphate level. Consequently, there is insufficient phosphated adenosine available to maintain the activity of the sodium‐potassium pump (Na^+^/K^+^ ATPase), which regulates the membrane potential and osmotic balance of cells. These disruptions can lead to severe electrolyte imbalances, cardiac failure and even death. Given this understanding, an exploration of the percentage of carbohydrate intake, rather than total daily energy intake, might show a more effective method in mitigating the risk of refeeding syndrome than reducing calories. Furthermore, considering that refeeding edema is associated with hyperinsulinemia, particularly concerning its effect on the natriuretic peptide inactivation and capillary leakage (da Silva et al. 2020), it is essential to address these factors in the analysis.

Additionally, to address the pathomechanism, it would be more scientifically robust to clearly define and diagnose the edema, systematically calculate nutritional and fluid intake, and explore relevant biochemical values. These should include monitoring electrolyte fluctuations during refeeding, assessing blood profiles, conducting urinalysis tests, and performing renal function assessments, as well as evaluating insulin resistance, for example, using the Homoeostatic Model Assessment of Insulin Resistance (HOMA‐IR). While the exact mechanisms underlying refeeding edema remain largely unclear, incorporating these tests and approaches would provide insights for a more comprehensive understanding of its pathomechanism. Monitoring electrolyte fluctuations would offer a clearer picture of the development of refeeding syndrome and its relation with edema, indicating how quickly and severely electrolyte imbalances may occur during refeeding. Moreover, blood profile assessments and urinalysis can help understand hemoconcentration or dehydration, which might serve as cofounding factors in fluid balance. Renal function tests and assessments of insulin resistance will further substantiate the hypothesis that refeeding edema could potentially arise from shifts in insulin sensitivity during the refeeding process in severely malnourished patients with restrictive EDs. By understanding these variables, clinicians can better tailor their approach in treating patients, ensuring adequate nutrition and fluid intake, optimizing recovery, and minimizing risks associated with refeeding.

In summary, I really appreciate the authors' work in this paper and hope this commentary encourages a more critical approach to understanding refeeding edema in patients with restrictive EDs. I look forward to future research in this area, as it is important for providing a clearer understanding of refeeding edema, allowing for a personalized approach and ensuring patient safety during refeeding treatment.

## Conflicts of Interest

The author declares no conflicts of interest.
